# Prevalence and multilocus genotyping of *Giardia duodenalis* in dairy calves in Xinjiang, Northwestern China

**DOI:** 10.1186/s13071-016-1828-3

**Published:** 2016-10-13

**Authors:** Meng Qi, Haiyan Wang, Bo Jing, Rongjun Wang, Fuchun Jian, Changshen Ning, Longxian Zhang

**Affiliations:** 1 College of Animal Science and Veterinary Medicine, Henan Agricultural University, 95 Wenhua Road, Zhengzhou, 450002 People’s Republic of China; 2 College of Animal Science, Tarim University, 1487 Tarim Road, Alar, 843300 People’s Republic of China; 3 Department of Animal Science, Henan Vocational College of Agriculture, Zhongmu, 451450 People’s Republic of China

**Keywords:** *Giardia duodenalis*, Prevalence, Genotyping, Dairy calves

## Abstract

**Background:**

*Giardia duodenalis* is an important protozoan parasite. It is an established zoonotic pathogen and dairy calves have been implicated as one of the most important sources of human infection. This study was conducted to assess the prevalence and multilocus genotyping of *G. duodenalis* in dairy calves in the Xinjiang Uyghur Autonomous Region, northwestern China.

**Findings:**

A total of 514 fresh fecal samples were randomly collected from dairy calves in 15 farms in Xinjiang, 13.4 % (69/514) tested positive for *G. duodenalis* by polymerase chain reaction (PCR) detection of the small subunit ribosomal RNA (SSU rRNA) gene, with the prevalence being 9.7 % (23/237) and 16.6 % (46/277) in pre- and post-weaned calves, respectively. Sequence analysis of the SSU rRNA gene predominantly detected *G. duodenalis* assemblage E (92.8 %, 64/69), whereas assemblage A was identified in five samples (7.2 %, 5/69). All *G. duodenalis*-positive samples were assayed with PCR followed by sequencing at β-giardin (*bg*), glutamate dehydrogenase (*gdh*) and triosephosphate isomerase (*tpi*) genes, and 29, 37 and 33 sequences were obtained, respectively. The presence of mixed *G. duodenalis* assemblage A and E was detected in only one sample. Multilocus genotyping yielded 15 multilocus genotypes (MLGs), one new assemblage A MLG, and 14 assemblage E MLGs. All assemblage E MLGs identified here differed genetically from those of cattle from Henan Province, Central China.

**Conclusions:**

Our data indicate that *G. duodenalis* is a common parasite in dairy calves in Xinjiang, China, and calves appear to be a reservoir of *G. duodenalis* that is infectious to humans. The differences in the distribution of *G. duodenalis* assemblage E MLGs from cattle were likely to be because of geographical segregation.

## Background

*Giardia duodenalis* (syns *G. lamblia*, *G. intestinalis*) is one of the most frequent enteroparasites worldwide with a broad host range that includes humans, livestock, companion animals and wildlife [1]. *Giardia* cysts commonly occur in the aquatic environment and transmission of *Giardia* cysts to humans occurs mainly through indirect routes such as contaminated surface water or foods or through direct contact with infected individuals [2]. Cattle are considered as a source of waterborne outbreaks of giardiosis in humans because of the reported high prevalence of *G. duodenalis* infection combined with the large output of feces, potentially leading to contamination of surface and ground water [3, 4].

*Giardia duodenalis* consists of at least eight genetically different assemblages, A–H, of which assemblages A and B infect both humans and other mammals, while the remaining assemblages (C–H) appear to be host-specific [5]. Surveys of dairy cattle worldwide have reported predominant prevalence of assemblage E, followed by the zoonotic assemblages A and B [1, 6]. Recently, feline-specific assemblage F was found in asymptomatic adult cattle in northern Spain [7]. *Giardia duodenalis* infections in adult dairy cattle are generally lower than in calves, but calves were more frequently infected with zoonotic assemblages A and B compared with assemblage E [8–11]. A study in New Zealand identified assemblages A and B in 40 *G. duodenalis* isolates from calves and from 30 humans living in the same region; the isolates were collected over a similar period, suggesting that calves posed a great risk of *G. duodenalis* infection to humans [12].

Little is known about the prevalence of *G. duodenalis* in calves in China, and current data on the assemblage distribution and multilocus genotyping of *G. duodenalis* in dairy calves remain unclear. In the present study, fecal samples from dairy calves were collected in the Xinjiang Uyghur Autonomous Region were analyzed for the presence of *G. duodenalis* using polymerase chain reaction (PCR) of the small subunit ribosomal RNA (SSU rRNA) gene. All *G. duodenalis*-positive samples were characterized for β-giardin (*bg*), glutamate dehydrogenase (*gdh*) and triosephosphate isomerase (*tpi*) genes to elucidate *G. duodenalis* genotypes.

## Methods

### Study area and sample collection

From August to September 2013, a total of 514 fecal samples consisting of 237 from pre-weaned calves (0–60 days) and 277 from post-weaned calves (61–150 days) were randomly collected from 15 intensively reared dairy cattle farms near the cities of Wujiaqu, Changji, Urumqi, Korla, Tacheng, Zhaosu and Aksu in Xinjiang Uyghur Autonomous Region (73°40'E–96°18'E, 34°25'N–48°10'N), northwestern China (Table 1). The farms are among the largest dairy farms aslo ranked among the top producing dairy farms in the region, consisting of 200–5,000 animals per farm. Before the sampling, we did not have data about the epidemiological situation of the farms. The farms were visited on a single occasion and the fecal samples were randomly collected from 20–30 % of the animals. The pre-weaned calves were bred in different calf hutch, respectively. The post-weaned calves were intensively reared in different stalls, with 10–30 calves per stall. At the time of fecal collection, no apparent diarrhea was seen in the herds. Fecal samples were collected directly from the rectum using disposable gloves and plastic containers. Fecal samples were maintained at 4 °C before DNA extraction.

**Table 1:** Prevalence of *G. duodenalis* and assemblages determined by sequence analysis of the SSU rRNA, *bg*, *gdh* and *tpi* genes

Farm	No. of samples	No. of positive for *G. duodenalis* (%) [95 % CI]	SSU rRNA gene (*n*)	*bg* (*n*)	*gdh* (*n*)	*tpi* (*n*)
WujiaquA	18	0				
WujiaquB	31	1 (3.2) [0–7.6]	E (1)		E (1)	E (1)
WujiaquC	20	3 (15.0) [6.2–23.8]	E (2), A (1)	E (1), A (1)	A (1)	A (1)
Changji	33	1 (3.0) [0–7.2]	A (1)			A (1)
Urumqi	32	3 (9.4) [3.8–15.0]	E (3)	E (1)	E (1)	
KorlaA	11	1 (9.1) [0–21.5]	E (1)			
KorlaB	13	1 (7.7) [0–18.3]	E (1)			
KorlaC	32	2 (6.3) [1.2–11.4]	E (1), A(1)	A (1)	E (1), A (1)	E (1), A (1)
KorlaD	33	2 (6.1) [1.1–11.1]	E (1), A(1)			
Tacheng	8	0				
Zhaosu	8	1 (12.5) [0–29.5]	A (1)		A (1)	A (1)
AksuA	48	9 (18.8) [14.0–23.6]	E (9)	E (8)	E (7)	E (6)
AksuB	58	7 (12.1) [8.3–15.9]	E (7)	E (2)	E (3)	E (2)
AksuC	70	17 (24.3) [20.5–28.1]	E (17)	E (8)	E (12)	E (11)
AksuD	99	21 (21.2) [18.3–24.1]	E (21)	E (7)	E (9)	E (7), A (1)
Total	514	69 (13.4) [12.6–14.2]	E (64), A (5)	E (27), A (2)	E (34), A (3)	E (28), A (5)

*Abbreviations*: *CI* 95 % confidence interval, *A* assemblage A, *E* assemblage E

### DNA extraction and PCR amplification

Genomic DNA was extracted from all fecal samples using the E.Z.N.A.R^®^ Stool DNA Kit (Omega Biotek, Norcross, GA, USA) according to the manufacturer’s instructions. For screening *G. duodenalis*, previously described nested PCR assays were used to amplify the SSU rRNA gene [13]. Because there is no variability in the SSU rRNA gene among *G. duodenalis* assemblages, we analyzed multilocus sequence polymorphisms based on β-giardin (*bg*) [14], glutamate dehydrogenase (*gdh*) [15] and triose phosphate isomerase (*tpi*) [16] genes to determine *G. duodenalis* subtypes. DNA from all *G. duodenalis*-positive samples were subjected to further PCR analysis of the *bg*, *gdh* and *tpi* genes according to previously described nested PCR protocols [14–17].

### Sequence analysis

PCR amplicons were sent to Beijing Nuosai Biological Engineering Biotechnology Company for bi-directional sequencing on an ABI PRISM™ 3730 XL DNA Analyzer using the BigDye Terminator v3.1 Cycle Sequencing Kit (Applied Biosystems, Foster City, CA, USA). Sequences were identified by alignment with reference sequences downloaded from GenBank using MEGA 5 software (http://www.megasoftware.net/). To study the relationship between different isolates in more detail, phylogenetic analyses were performed using a concatenated dataset of *bg*, *gdh* and *tpi* gene sequences with the multilocus genotypes (MLGs) of *G. duodenalis.* The reference MLGs of *G. duodenalis* in cattle from Henan Province, Central China originate from a previous study [17]. The nucleotide neighbor-joining phylogenetic trees were based on the Tamura-Nei model. The reliability of these trees was assessed by bootstrap analysis with 1,000 replicates. Nucleotide sequences obtained in this study have been deposited in the GenBank database under accession numbers KT369759–KT369788.

### Statistical analysis

The Chi-square test was used to compare *G. duodenalis* infection rates between age groups. Differences were considered statistically significant when *P* < 0.05.

## Results and discussion

*Giardia duodenalis* infections have been frequently reported in calves and the prevalence of *G. duodenalis* in fecal samples from calves has shown wide variation (0–100 %) [5, 8, 10, 13, 17–27]. In the present study, 69 samples were positive for amplification of the SSU rRNA gene and the overall prevalence for *G. duodenalis* was 13.4 % (69/514) (Table 1). The overall prevalence was lower than that previously reported in Belgium (22 %, 110/499) [8], Australia (26.9 %, 98/364) [10], Norway (49 %, 679/1386) [21], and Europe (Germany, UK, France, and Italy; 45.4 %, 942/2072) [23], and higher than that in Germany (7.2 %, 112/1564) [25]. However, it is difficult to compare prevalence data, which are influenced by a range of factors, including the diagnostic method and study design, geographical conditions, age of animals, number of samples from each farm, total number of samples and sampling season.

There was a significant association of *G. duodenalis* infection with the age of animals. In the present study, the prevalence in post-weaned calves (16.6 %) was higher than the prevalence in pre-weaned calves (9.7 %) (Table 2). Chi-square testing showed that *G. duodenalis* prevalence was significantly different between the two age groups (*χ* ^2^ = 5.23, *df* = 1, *P* = 0.022). This is similar to reports from Canada [18], Norway [21], United States [19, 20] and Germany [25]. In contrast, other authors reported pre-weaned calves in Henan Province, Heilongjiang Province, Jilin Province, Liaoning Province, Shaanxi Province and Ningxia Hui Autonomous Region, China to have the highest prevalence [11, 17, 26–28]. These differences may be the result of different diagnostic modalities or varying environmental, geographical, or management factors.

**Table 2:** Prevalence of *Giardia duodenalis* and distribution of assemblages by age

Age	No. of samples	No. of positive for *G. duodenalis* (%) [95 % CI]	SSU rRNA gene (*n*)	*bg* (*n*)	*gdh* (*n*)	*tpi* (*n*)
Pre-weaned	237	23 (9.7) [8.4–11.0]	E (20), A (3)	E (9), A (1)	E (10), A (2)	E (9), A (3)
Post-weaned	277	46 (16.6) [15.3–17.9]	E (44), A (2)	E (18), A (1)	E (24), A (1)	E (18), A (2)

*Abbreviations*: *CI* 95 % confidence interval, *A* assemblage A, *E* assemblage E

Sequence analyses of the amplified SSU rRNA gene fragments were successful for all 69 PCR-positive samples, five of which were *G. duodenalis* assemblage A (7.2 %, 5/69) and the rest were assemblage E (92.8 %, 64/69) (Table 1). The genetic diversity of these positive *G. duodenalis* isolates was determined by amplification and sequencing of the *bg*, *gdh* and *tpi* genes, with 29 *bg*, 37 *gdh* and 33 *tpi* gene sequences being obtained (Table 1). Only one isolate (XJ1680) was identified as assemblage E by its SSU rRNA gene sequence but as assemblage A by its *tpi* gene sequence. These findings are similar to those in previous reports of calves from Canada [9], Australia [10], China [17, 26, 27], United States [19, 20, 22], Belgium [23] and Germany [29]. In China, *G. duodenalis* assemblage B was also found in calves from Ningxia Hui Autonomous Region [11] and Heilongjiang Province [28].

A comparison of the *G. duodenalis* assemblages between the age groups is presented in Table 2. Both assemblages, A and E, of *G. duodenalis* were detected in pre- and post-weaned calves in the present study, which is consistent with other studies in Henan and Shaanxi Province, China [17, 27], United States [19, 20] and Europe [23]. However, there was a higher prevalence of assemblage A in pre-weaned calves than was observed in this study. While assemblages B and E of *G. duodenalis* were detected in pre-weaned calves, only assemblage E was detected in post-weaned calves in Ningxia Hui Autonomous Region, China [11].

**Fig. 1:**
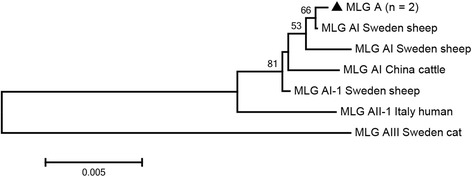
Phylogenetic relationships between *Giardia duodenalis* assemblage A multilocus genotypes (MLGs). The phylogenetic tree was constructed using a concatenated dataset of *bg*, *tpi* and *gdh* gene sequences, and the neighbor-joining method analysis resulted in identical topologies. Isolates from the present study are indicated by black triangles

**Fig. 2:**
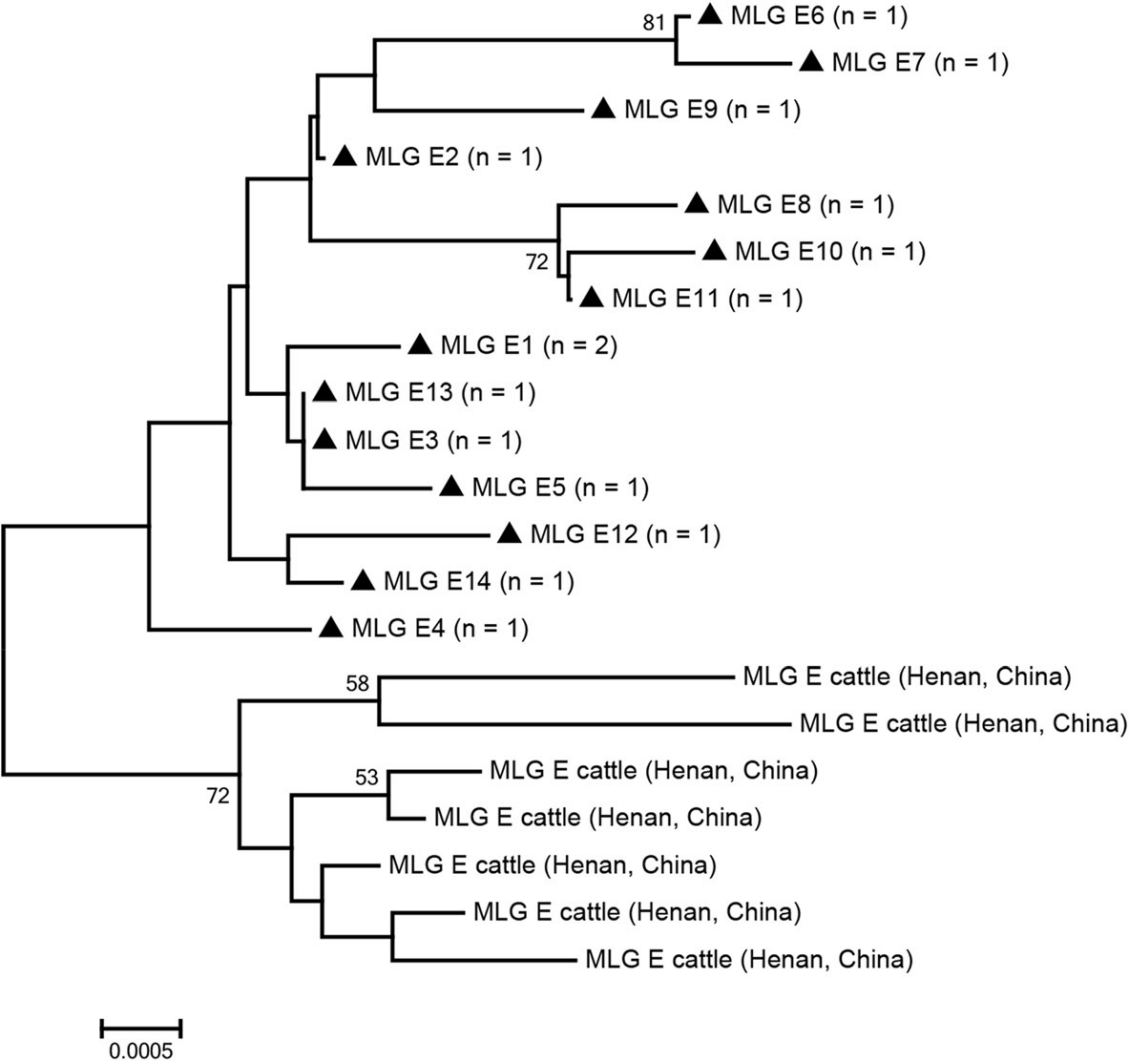
Phylogenetic relationships between *Giardia duodenalis* assemblage E multilocus genotypes MLGs. The phylogenetic tree was constructed using a concatenated dataset of *bg*, *tpi* and *gdh* gene sequences, and the neighbor-joining method analysis resulted in identical topologies. Isolates from the present study are indicated by black triangles

Based on multilocus genotyping, the MLG model was used to better understand the characteristics of *G. duodenalis* in humans and animals from different geographic regions, which is helpful for unveiling zoonotic potential and dynamic transmission [2, 30]. In the present study, of the 29 isolates successfully sequenced for *bg* gene, two were identified as one assemblage A sequence, while 27 were identified as seven assemblage E sequences (Table 3). For the *gdh* gene, of the 37 *G. duodenalis* isolates successfully sequenced, three were identified as one assemblage A sequence, while 34 were identified as 11 assemblage E sequences (Table 3). For the *tpi* gene, of the 33 *G. duodenalis* isolates successfully sequenced, five were identified as two assemblage A sequences, while 28 were identified as eight assemblage E sequences (Table 3). All three genes were successfully amplified and sequenced from 17 isolates, one or two genes were amplified from 31 isolates, while the remaining 21 samples were repeatedly negative (Table 3). The 17 isolates that were successfully genotyped at all three genes formed one assemblage A MLG and 14 different assemblage E MLGs (Table 3). In the present study, one assemblage A MLG from two calf isolates was identified as a novel MLG A (Fig. 1). Whether this MLG A has zoonotic potential requires systematic molecular epidemiological investigations in humans and animals. For assemblage E, phylogenetic analysis showed all assemblage E MLGs clustered broadly with previously reported cattle isolates from Henan Province, Central China (Fig. 2). Meanwhile, of the 22 assemblage E MLGs were detected in dairy calves and Qinchuan calves in Shaanxi Province, China, none of MLGs was identical to the results in Henan Province [17, 27]. These findings suggest that there might be geographical distribution differentiation among isolates.

**Table 3:** Multilocus characterization of *Giardia duodenalis* isolates based on *bg*, *gdh* and *tpi* genes

Calf ID	Genotype (GenBank accession no.)			MLG type (*n*)
	*bg*	*gdh*	*tpi*	
XJ214, XJ646	A (KT369769)	A (KT369777)	A1 (KT369759)	MLG A (1)
XJ224			A1	
XJ1109		A	A1	
XJ1680			A2 (KT369760)	
XJ133, XJ1348		E1 (KT369778)	E1 (KT369761)	
XJ211, XJ1704	E1 (KT369770)			
XJ442	E2 (KT369771)	E2 (KT369779)		
XJ631		E3 (KT369780)	E2 (KT369762)	
XJ1226, XJ1234	E3 (KT369772)	E4 (KT369781)	E2	MLG E1 (2)
XJ1233	E3	E5 (KT369782)		
XJ1237	E3	E1	E3 (KT369763)	MLG E2 (1)
XJ1239	E4 (KT369773)		E4 (KT369764)	
XJ1250		E6 (KT369783)		
XJ1260	E3	E1	E2	MLG E3 (1)
XJ1261, XJ1457	E3		E2	
XJ1263	E5 (KT369774)	E1		
XJ1349	E3	E1		
XJ1353	E6 (KT369775)	E5	E2	MLG E4 (1)
XJ1469		E7 (KT369784)	E3	
XJ1483	E3	E8 (KT369785)	E2	MLG E5 (1)
XJ1488	E3	E5	E5 (KT369765)	MLG E6 (1)
XJ1490	E3	E5	E6 (KT369766)	MLG E7 (1)
XJ1492		E8	E3	
XJ1493, XJ1656, XJ1676, XJ1709		E1		
XJ1500		E9 (KT369786)		
XJ1501	E3	E4	E7 (KT369767)	MLG E8 (1)
XJ1504		E5		
XJ1506	E3	E1	E8 (KT369768)	MLG E9 (1)
XJ1509	E3	E10 (KT369787)	E7	MLG E10 (1)
XJ1510	E3	E1	E7	MLG E11 (1)
XJ1516, XJ1670			E2	
XJ1647	E3	E11 (KT369788)		
XJ1675		E1	E7	
XJ1681	E2		E2	
XJ1682	E7 (KT369776)	E1	E1	MLG E12 (1)
XJ1689	E3	E1	E2	MLG E13 (1)
XJ1693	E2			
XJ1699		E5	E1	
XJ1706	E1	E1	E2	MLG E14 (1)

## Conclusion

The results of the present study confirm previous findings in other areas of China that *G. duodenalis* infections are common in dairy calves. The livestock-specific *G. duodenalis* assemblage E was the predominant assemblage, but the zoonotic assemblage A was also present in Xinjiang, China. The differences in the distribution of *G. duodenalis* assemblage E MLGs from cattle likely indicate a geographical segregation. Moreover, more multilocus genotyping studies are needed, which may help to identify polymorphisms and to elucidate the zoonotic potential of *G. duodenalis*.

## Abbreviations

*bg*: β-giardin
*gdh*: Glutamate dehydrogenase
MLGs: Multilocus genotyping
PCR: Polymerase chain reaction
SSU rRNA: Small subunit ribosomal RNA
*tpi*: Triosephosphate isomerase
